# Criminals: How to Make and How to Mend Them

**Published:** 1891-01-17

**Authors:** 


### Criminals: How to Make and How to Mend Them.

A criminal is, speaking philosophically, simply a creature whose ideas and practice are opposed to those of the society in which he lives. This may seem to be a somewhat mealy-mouthed definition, but it is the only one large enough to cover the whole class. The saint or hero, the martyr to creed or country, to whom a nation may now be raising statues was simply, in the eyes of his judges, a criminal, a lawbreaker. As, however, the majority of mankind are pretty well agreed as to what things are undesirable in social custom, it is easy to discuss criminals, their crimes and punishments, without being caught up by some reference to the martyrs of the past, or even the political offenders of a nearer date. No one will justify theft and murder except the thieves and murderers themselves.

These, however, will and do justify their acts. The professional thief—the aristocrat of the criminal classes—regards himself as the poor but comparatively honest rival of tradesmen, stockbrokers, and all others who, from his point of view, are aided in their chicanery by the law that punishes him.

> "Treason can never prosper. What's the reason?
> Because whene'er it prospers 'tis not treason."

So says the satirist regarding political crimes, and the robber applies the principle to moral offences. This is the argument, the life-philosophy of the most intelligent criminal, and, in a more or less conscious form, it permeates the whole class, down to those moral lunatics to whom it seems a sufficient explanation of the vilest crimes that they wanted something not conveniently to be obtained in any other way. Mr. Havelock Ellis has collected all manner of information regarding criminals into his compact little book*, and a perusal of it leaves little doubt that in almost every case crime is the result of an absolute lack of perception of any real difference between right and wrong. The causes of this dulness are still obscure, though the researches of Lombroso, Marro, Despine, Maudsley, and others in criminal anthropology show a frequent connection between the moral flaw and some peculiarity in physical development. Benedikt, of Vienna, has observed in the criminal brain frequent confluence of the fissures and an additional convolution of the frontal lobe, which thus resembles that of the carnivorous animals. This may account for much instinctive brutality, while many crimes due to apparently meaningless malice might be traced to the fact, proved by post-mortem examinations, that a large proportion of criminals suffer from meningitis. Lombroso sets down the number as about fifty per cent. of the whole, and other forms of physical degeneration are common.

Crime may thus be regarded as a disease, and most scientists would gladly see it treated as such, though there will inevitably be much divergence of opinion as to the treatment. It is not likely, and we venture to think it would be most undesirable, that either public or judicial opinion should go as far with us as it does in Russia, where a young girl who had murdered a child in order to secure to her lover the inheritance of the child's father's money was acquitted, because Professor Babruski decided that, though not mad, she was devoid of moral notions, and being incurable would not be cured by seclusion in an asylum. A young woman who is "devoid of moral notions" may commit a similar act another time, and seclusion of some kind is desirable, if not for her own sake, for that of her possible victims. The hero of Tolstoi's "Kreutzer Sonata" is evidently a creature of this kind, and whatever the author may think, we incline to the old Scotch judge's belief that he "would be nane the waur o' a hanging." That may not be the best thing one can do with him, but it is better than letting him go about dealing death wherever his fevered brain may think it is deserved. In that way "Jack the Ripper" is evolved.

These people, morally deficient in the highest degree, are sometimes intellectually gifted beyond the average. Thomas Wainewright, essayist, forger, and murderer, one of the most famous criminals of the early part of the century, was possessed of the most sensitive and refined literary tastes. He poisoned his uncle, his sister-in-law, a friend in whose house he was staying—always after insuring their lives for his benefit—but he complained of the coarseness of Hazlitt's writing, he told how "his blessed art touched her renegade" after a period of dissipation, and he shed tears of happiness over Wordsworth's poems. When he was sentenced to transportation for life he was shocked that his gaolers put him in irons. "They think me a desperado!" he exclaimed. "Me! the companion of poets, philosophers, artists, and musicians, a desperado!" He absolutely felt himself ill-used by such treatment. A little German girl, Marie Schneider, is another example of what is termed the instinctive criminal When only twelve years old she killed a girl of three who wore little gold ear-rings. She wanted those ear-rings to sell, that she might buy cakes with the money. The whole matter seemed to her quite simple and natural, and when questioned before the judge she told the story clearly and intelligently, as if indeed she were rather pleased at giving such lucid answers. Yet she knew what murder was; she knew that she was a murderess. She knew, too, that she would not be executed for her crime, because she was still too young; and she resented the dry bread they gave her to eat in the prison—and she so fond of cakes! Neither the man nor the child felt any regret for their sins; they could hardly realize others' pain, while they were acutely sensible to personal discomfort. People of this temperament are practically incurable.

The constitution of society, to which some people are ready to refer every evil under the sun, can hardly be held responsible for such sins as these. One cannot imagine the society in which Marie Schneider will not hunger for cake nor Wainewright thirst for champagne; but our social ideas, right in themselves, are sometimes indirectly responsible for crime, and sometimes our methods of punishment encourage it. What does a fortnight's imprisonment do for the habitual drunkard? It restores his strength after the burst of intoxication that brought him before the magistrate, and invigorates him for the fresh one he will have when he goes out. When one reads of prisoners with twenty or fifty such convictions recorded against them, one feels that the futility of these short periods of imprisonment as serving either as deterrent or cure is demonstrated to perfection.

But the longer periods of detention that await theft and forgery are not in any sense more beneficial to the prisoner, and therefore more profitable to society. Your thief is shut up for five years, during which time he thinks sadly of the way in which he bungled that last job, and meditates on new and improved methods of robbery. Look after him as well as you may, he will find means to communicate with his fellow-prisoners, and their intercourse is sure to breed mischief. Even if solitary confinement could be enforced, it is, as a remedial measure, of little use. Prince Krapotkin and Mr. Michael Davitt have spoken of its depressing effects, and they are men of intelligence, men of resources. What its effects are on the common sort can be seen in the mutinies, the malingering, the obscene descriptions scribbled on the walls of cells, of which all prison officials can tell us. The average prison does very little to reform the criminal, and keeps him out of mischief only so long as its walls surround him. What

* "The Criminal," by Havelock Ellis. Contemporary Science Series. (London: Walter Scott, 24, Warwick Lane, Paternoster Row.)

must be sought, in the name both of Christianity and civilization, is a truly remedial discipline.

The prison at Elmira, in the State of New York, points out the way to the civilized world. Its intellectual and manual training have already done wonders. Only 15 per cent. of its inmates are known to return to criminal courses; but then they have been taught a trade during their imprisonment, and —even more important—a gradual increase of liberty, granted as it is earned, has taught them self-respect and self-control, and fitted them for some other than the criminal's place in the outer world. A still more remarkable prison exists in Japan, where criminals produce *cloisonné* enamel, wood-carving, and pottery; but it is doubtful if our British thieves and burglars have artistic taste for that. But the indeterminate sentence (already treated of in these columns) by which it depends largely on the prisoner's own conduct when he regains his liberty, is perfectly practicable anywhere. "Conduct" should not, in the criminal's own interest, mean mere submission to the dictates of warders, governors, and chaplains, but the development of such intelligence and industry as will fit him to hold his own among honest men. This cannot be done quickly, for at best the criminal is not a favourable subject, and often the ductile years of youth are past. But if in even a few it arouses the dormant intelligence, and teaches how interesting as well as profitable work can be, it is surely better worth trying than all the cranks and tread-mills that ever were invented.

